# The impact of parental migration on health status and health behaviours among left behind adolescent school children in China

**DOI:** 10.1186/1471-2458-10-56

**Published:** 2010-02-03

**Authors:** Yang Gao, Li Ping Li, Jean Hee Kim, Nathan Congdon, Joseph Lau, Sian Griffiths

**Affiliations:** 1 School of Public Health and Primary Care, the Chinese University of Hong Kong, Hong Kong SAR, China; 2 Injury Prevention Research Center, Medical College of Shantou University, Shantou, China; 3 Department of Ophthalmology, Faculty of Medicine, the Chinese University of Hong Kong, Hong Kong SAR, China; 4 Joint Shantou International Eye Centre, Shantou, China

## Abstract

**Background:**

One out of ten of China's population are migrants, moving from rural to urban areas. Many leave their families behind resulting in millions of school children living in their rural home towns without one or both their parents. Little is known about the health status of these left behind children (LBC). This study compares the health status and health-related behaviours of left behind adolescent school children and their counterparts in a rural area in Southern China.

**Methods:**

A cross-sectional study was conducted among middle school students in Fuyang Township, Guangdong, China (2007-2008). Information about health behaviours, parental migration and demographic characteristics was collected using a self-administered questionnaire. Overweight/obesity and stunting were defined based on measurements of height and weight. Univariate and multivariate analyses were used to estimate the differences in health outcomes between LBC and non-LBC.

**Results:**

18.1% of the schoolchildren had one or both parents working away from home. Multivariate analysis showed that male LBC were at higher risk of skipping breakfast, higher levels of physical inactivity, internet addiction, having ever smoked tobacco, suicide ideation, and being overweight. LBC girls were more likely to drink excessive amounts of sweetened beverage, to watch more TV, to have ever smoked or currently smoke tobacco, to have ever drunk alcohol and to binge drinking. They were also more likely to be unhappy, to think of planning suicide and consider leaving home.

**Conclusions:**

Our findings suggest that parental migration is a risk factor for unhealthy behaviours amongst adolescent school children in rural China. Further research is required in addition to the consideration of the implications for policies and programmes to protect LBC.

## Background

China has been undergoing rapid urbanization in the last few decades. By 2000, over 100 million people in China were migrants [1]. The majority of internal migrants are adults aged 25-49 years old, moving from rural to urban areas to seek employment opportunities [2,3]. Children often do not move with their parents due to financial constraints and the transient nature of the work in urban areas [1]. The term "left behind children (LBC)" is ascribed to the children staying in their rural home towns and villages. By 2000, the number of LBC living in rural areas across China was estimated to be around 20 million, and about 10% of the LBC population in China live in Guangdong province, which ranks the second highest amongst the 31 provinces in China [1].

Unhealthy behaviours such as smoking tobacco and poor diet during adolescence perpetuate into adulthood and have lasting health impacts [4,5]. Evidence from studies in various countries suggests that a stable family environment contributes to healthy development among children [4-10]. Mixed results have however been generated with respect to the health impact of parental migration on the health of LBC [11-16]. Negative impacts such as substance use and poorer psychosocial health have been documented [11-13]. However, some Asian studies (including those conducted in India, Pakistan, Sri Lanka, Philippines, and Thailand) have indicated that the actual impact of parental migration on educational and psychological aspects of the LBC is only modest [14-16].

In China studies have reported that LBC encounter more educational problems, such as higher rates of absence and dropout rates [17,18]. LBC are also found to be more vulnerable to psychological problems such as loneliness, depression, anxiety, and introversion [19-22]. One study however, found no significant difference in academic performance between LBC and non-LBC living in three Chinese provinces [23]. There is a dearth of data concerning physical health of LBC in China.

In this study, we investigated the impact of parental migration among rural middle school students in Southern China, and explored their nutritional health status (indicated by stunting and overweight/obesity) and health-related behaviours (including eating habits, physical activity, smoking, drinking, internet abuse, and emotional issues). Through comparison with their counterparts in the same rural area whose parents had not migrated we were able to describe the impact of left behind status.

## Methods

### Subjects

A cross-sectional study was conducted between December 2007 and June 2008 in Fuyang Township, Guangdong Province. Fuyang has a population of 87,134 and a per capita annual net income of 5,154 RMB (2008), which compares well with national and provincial mean figures of 4,140 RMB and 5,624 RMB respectively [24,25]. The township is categorized as a Type II rural area (Type I is very affluent and Type IV is very poor). There are five junior secondary schools (1 vocational + 4 regular) for children aged 13-15 years old and one senior secondary school for those aged 16-18 years. All Years 1 and 2 junior and senior secondary school students were invited to join the study. Field investigators assisted students to complete a questionnaire in their classrooms without the presence of teachers. Height and weight were measured in a designated room in each school by two investigators using calibrated scales. All investigators received training in advance. Informed written consent was obtained from the students in advance. Ethics approval was obtained from the Ethics Committees at the Chinese University of Hong Kong.

### Measures

#### Definition of LBC cases

In order to be consistent with the national definition of "floating population", this study adopted Zhou and Duan's definition of LBC as "children under 18 who have been left behind at their original residence while one or both parents migrate into other places for work and have been not living together with them for at least six months" [18]. Students were asked: 1, "Has your father/mother NOT been living with you for at least six months in a row?" and 2, "Why has your father/mother NOT been living with you?". Children who responded "No" to the first question and gave "working" as the reason of separation were identified as LBC. Separation due to divorced/separated or deceased parents did not constitute LBC cases.

#### Socio-demographic characteristics

Information about students' ethnicity, gender, age, class, grade, school, whether boarding in the school, as well as education level and occupation of parents, whether living with grandparents, housing type, and annual family income per capita was collected.

#### Health-related behaviours

The questions were adapted from those being used in the 2005 Chinese Youth Risk Behaviour Survey (YRBS), the 2007 YRBS of the U.S., and the Global School-based Student Health Survey (GSHS) to develop the questionnaire [26-28]. Questions were related to eating habits, physical activity, sedentary behaviours, internet use, tobacco use, alcohol use, and mental and emotional health. Being physically active was defined as reporting being active at least 60 minutes per day, including the time they spent in any kind of activity that increased their heart rate and made them breathless some of the time [26]. Binge drinking was defined as "ever having had at least five glasses of alcohol within one or two hours in the past 30 days" [26]. Internet addiction was identified using the Youth 8-item Internet Addiction Scale [29].

#### Nutritional status

Height and weight were measured and body mass index (BMI, kg/m^2^) was derived to indicate students' nutritional status. Height (recorded to the nearest 1 cm) was measured with bare or stockinged feet. Weight (recorded to the nearest 0.1 kg) was measured with the subject lightly dressed. Overweight and obese children were defined according to the Chinese BMI-for-Age cut-off points for children aged 7-18 years old [30]. The World Health Organization (WHO) definition of < -2SD of Height-for-Age Z scores was used to identify stunted children [31].

### Data analysis

Data was entered through the Remark Office OMR^® ^6 (Gravic Inc., Malvern, PA). SPSS for Windows 16.0 (SPSS Inc., Chicago, IL) was used for all statistical analyses. Analyses were stratified by gender. To test statistical significance of between-group differences (e.g. LBC versus non-LBC), chi-square and One-way Analysis of Variance tests were used; Odds Ratio (OR) and Mean Difference (MD) and respective 95% Confidence Intervals (95% CI) were also derived. Multiple logistic regression and linear regression models were fitted, adjusting for potential confounders including age (adjusted as a continuous variable), ethnicity, school type (regular vs. vocational school), whether living in a boarding school, parental education level, whether living with grandparents, housing type, and whether having computer access at home [32,33]. We did not adjust for parents' occupation because migrant parents of LBC were non-farmer and the health impact due to parental migration would be removed if it is adjusted [32,33]. Significance and goodness of fit were tested for each final model [32,33].

## Results

### Participation

One school was excluded due to lack of interest in participation. This school is a regular junior school and has similar characteristics with the other schools in the study. 5 schools took part in the final study. A total of 3226 students were invited to take part in the study, 3141 (97.4%) agreed, and 2986 (92.6%) completed the questionnaire and the measurements and were included in the analysis (91.5% for boys and 94.2% for girls respectively).

### Socio-demographic characteristics

18.1% (541) of the respondents were LBC. 51.4% were male and their ages ranged from 10-18 years (mean age = 14.2 years, median = 14.0 years). When compared with non-LBC, LBC were more likely to be boys, Year 1 junior secondary school students and their average age was 13.2 years (SD = 0.63 years). They were more likely to be from an ethnic minority, and not to be a boarder (Table 1, p < 0.05). In addition, parents of LBC were less likely to be farmers and their mothers were more likely to be highly educated. LBC were less likely to have a computer and internet access at home (p < 0.05). Similar proportions of LBC and non-LBC (69.2% vs. 70.9%, P > 0.05) were unaware of their yearly family income per capita and another 13.5% refused to answer the question (data not shown), possibly because they did not know.

**Table 1:** Comparison of demographic characteristics between LBC^a ^and non-LBC

	Total (N = 2986)	Non-LBC (N = 2445)	LBC (N = 541)	p-value (χ^2 ^test)
Gender (%)				0.075

Male	51.4	50.7	54.9	

Female	48.6	49.3	45.1	

Age *(year)*, Mean *(SD)*	14.2 *(1.4)*	14.3 *(1.5)*	13.7 *(1.2)*	< 0.001^b^

Ethnicity (%)				0.030

Han	99.4	99.5	98.7	

Others	0.6	0.5	1.3	

Grade (%)				< 0.001

Year 1 of junior secondary school	57.8	54.2	74.3	

Year 2 of junior secondary school	19.5	20.3	15.7	

Year 1 of senior secondary school	22.7	25.5	10.0	

Boarder in this term (%)				< 0.001

Yes	12.5	13.7	6.6	

No	87.5	86.3	93.4	

Father's education (%)				0.786

Primary school and below	24.7	24.5	25.9	

Junior secondary school	53.0	53.2	52.0	

Senior secondary school and above	22.3	22.3	22.1	

Father's occupation (%)				0.008

Farmer	31.9	33	26.5	

Non-farmer	68.1	67	73.5	

Mother's education (%)				0.005

Primary school and below	56.1	57.2	51.0	

Junior secondary school	34.1	33.7	35.9	

Senior secondary school and above	9.8	9.1	13.2	

Mother's occupation (%)				< 0.001

Farmer	47.6	50.2	35.4	

Non-farmer	52.4	49.8	64.6	

Living with grandparents (%)				0.608

Yes	25.9	25.7	26.8	

No	74.1	74.3	73.2	

House type (%)				0.352

Single-storey house	47.0	49.3	47.4	

Multi-storey house	53.0	50.7	52.6	

Having computer at home (%)				0.061

No	68.2	67.4	72.0	

Yes, but not connected with internet	12.9	13.0	12.7	

Yes, already connected with internet	18.8	19.6	15.3	

**a: LBC**: Left Behind Children;

**b**: t-test;

Totals for all categories may not sum to 2986 due to missing data.

### Comparison of health status and health-related behaviours between LBC and non-LBC

In general, LBC had a higher prevalence of all the unhealthy lifestyle behaviours such as smoking but lower rates of all other healthy behaviours with the exception of intake of fruits and milk and participation in sport teams (Table 2). A range of health outcomes (12 items for boys and 10 for girls) showed significance or were marginally significant before controlling for other factors, and 6 items for boys and 9 for girls reached at least marginal significance in adjusted models (Tables 3 &4). Overall, children with both parents absent were most likely to engage in risk behaviours (skipping breakfast, eating high-fat food and sweetened snacks, being physically inactive, spending more time watching TV, playing e-games and using internet, current smoking and drinking, and having been drunk). They were also most likely to have emotional and mental problems and to be overweight and stunted. Children with a migrant mother only were also more vulnerable compared to those with father's absence only.

**Table 2:** Comparison of health behaviours and physical health among LBC^a ^and non-LBC by gender

	Males			Females		
		
	All (n = 1536)	Non-LBC (n = 1239)	LBC (n = 297)	All (n = 1450)	Non-LBC (n = 1206)	LBC (n = 244)
**Dietary intake (%)**						

Felt hungry most of the time or always^1^	1.5%	1.2%	2.8%	1.1%	1.2%	0.6%

Ate breakfast everyday^2^	75.1%	76.3%	69.4%	73.4%	73.8%	71.1%

Ate vegetables ≥3 times/day^2^	61.0%	62.5%	53.3%	57.3%	58.0%	53.6%

Ate fruits ≥3 times/day^2^	39.4%	39.2%	40.6%	37.1%	36.0%	42.8%

Ate high-fat food ≥3 times/day^2^	15.9%	15.6%	17.8%	11.6%	11.2%	13.9%

Ate sweetened snack ≥3 times/day^2^	23.6%	23.2%	25.6%	23.1%	22.4%	27.1%

Drank sweetened beverage ≥3 times/day^2^	24.3%	24.4%	23.9%	11.9%	10.9%	17.5%

Drank milk ≥6 days/week^2^	7.6%	7.3%	9.4%	7.2%	7.2%	7.2%

**Physical activity & sedentary behaviours (%)**						

Physically active^§ ^≥4 days/week^2^	51.7%	53.3%	43.9%	46.6%	46.8%	45.8%

Participated in sports team(s)^ 3^	31.1%	30.7%	33.3%	23.7%	23.6%	24.1%

Watched TV ≥4 hours/day^2^	19.3%	19.4%	18.9%	18.7%	17.4%	25.9%

Did homework ≥4 hours/day^2^	11.5%	12.5%	6.1%	19.5%	20.1%	16.3%

Played electronic games ≥4 hours/day^2^	13.5%	13.0%	16.1%	5.1%	4.9%	6.0%

**Internet use (%)**						

Used internet^2^	60.2%	60.0%	61.1%	44.7%	45.7%	39.2%

Used internet ≥4 hours/day^2^	9.7%	9.5%	10.6%	3.9%	3.9%	4.2%

Internet addiction	8.4%	7.7%	11.7%	3.2%	3.3%	2.4%

**Tobacco & alcohol use (%)**						

Ever smoked	50.2%	49.3%	55.0%	10.8%	10.0%	15.1%

Currently smoked^1^	17.1%	16.3%	20.6%	1.0%	0.6%	3.6%

Ever drank alcohol	69.7%	69.1%	72.8%	42.7%	42.9%	41.9%

Currently drank alcohol^1^	33.6%	32.7%	37.8%	13.1%	12.9%	14.5%

Binge drinking^¶1^	12.1%	11.9%	13.3%	2.8%	2.1%	6.6%

Reported symptoms of drunkenness^3^	22.6%	22.4%	23.9%	10.2%	10.0%	11.4%

**Emotional and mental issues (%)**						

Felt lonely most of the time or always^3^	4.9%	4.6%	6.7%	6.9%	6.8%	7.8%

Felt unhappy most of the time or always^3^	13.8%	13.7%	14.4%	16.8%	16.3%	19.3%

Sleepless most of the time or always^3^	5.7%	5.4%	7.2%	6.3%	6.2%	6.6%

Felt sad or hopeless ≥2 weeks^3^	8.6%	8.3%	10.0%	10.3%	10.2%	10.8%

Suicide ideation^3^	6.3%	5.4%	11.1%	11.0%	10.3%	15.1%

Suicide plan^3^	1.3%	1.1%	2.2%	2.0%	1.6%	4.2%

Suicide attempt^3^	0.7%	0.6%	1.7%	1.2%	1.0%	2.4%

Ideation of leaving home^3^	14.0%	13.1%	18.3%	14.1%	13.3%	18.7%

Attempt of leaving home^3^	2.1%	2.0%	2.8%	0.7%	0.4%	2.4%

**Nutritional health (%)**						

Overweight	6.2%	5.2%	11.1%	4.7%	4.5%	5.4%

Obese	1.7%	1.6%	2.2%	0.9%	0.9%	1.2%

Stunted	6.2%	5.8%	8.3%	4.4%	4.5%	3.6%

	*Mean ± SD^4^*	*Mean ± SD^4^*	*Mean ± SD^4^*	*Mean ± SD^4^*	*Mean ± SD^4^*	*Mean ± SD^4^*

Height *(cm)*	160.5 ± *9.2*	161.0 ± *9.2*	158.3 ± *8.8*	153.7 ± *5.3*	153.9 ± *5.4*	152.9 ± *5.2*

Weight *(kg)*	47.9 ± *9.9*	48.1 ± *9.8*	47.1 ± *10.4*	44.2 ± *6.8*	44.4 ± *6.8*	43.2 ± 6.8

BMI *(kg/m^2^)*	18.4 ± *2.7*	18.4 ± *2.7*	18.6 ± *2.9*	18.7 ± *2.5*	18.7 ± *2.5*	18.5 ± *2.5*

**a: LBC**: Left Behind Children; Totals for all categories may not sum to1536 (for boys) or 1450 (for girls) due to missing data;

**1**: in the past 30 days; **2**: in the past 7 days; **3**: in the past 12 months; **4: SD**: Standard Deviation;

**§**: Physically active represents children who were physically active for a total of at least 60 minutes per day, including all the time they spent in any kind of physical activity that increased their heart rate and made their breaths hard some of the time;

^¶^Binge drinking: ever drank at least 5 glasses of alcohol within 1 or 2 hours in the past 30 day.

**Table 3:** Unadjusted and adjusted Odds Ratio (OR) of parental migration among boys (with either OR_unadj _or OR_mv _significant or marginally significant)

	Males (non-LBC = ref)					
	
	OR_unadj_	*(95% CI) *	p-value	OR_mv_	*(95% CI)*	p-value
**Dietary intake**						

Felt hungry most of the time or always^1^	2.35	*(1.07-5.15)*	**0.028**	1.31	*(0.43-3.95)*	0.633

Ate breakfast everyday^2^	0.75	*(0.56-0.99)*	**0.044**	0.75	*(0.53-1.05)*	**0.096**

**Physical activity & sedentary behaviours**						

Physically active^§ ^≥4 days/week^2^	0.61	*(0.47-0.78)*	**< 0.001**	0.67	*(0.50-0.92)*	**0.012**

Did homework ≥4 hours/day^2^	0.66	*(0.42-1.02)*	**0.062**	0.67	*(0.40-1.14)*	0.137

Played electronic games ≥4 hours/day^2^	1.35	*(0.96-1.90)*	**0.083**	1.41	*(0.93-2.16)*	0.110

**Internet use**						

Internet addiction	1.83	*(1.21-2.78)*	**0.004**	1.77	*(1.06-2.95)*	**0.029**

**Tobacco & alcohol use**						

Ever smoked	1.20	*(0.93-1.55)*	0.167	1.39	*(1.02-1.90)*	**0.037**

Currently smoked^1^	1.31	*(0.96-1.80)*	**0.093**	1.37	*(0.92-2.05)*	0.118

**Emotional and mental issues**						

Felt sad or hopeless ≥2 weeks^3^	1.89	*(1.20-2.97)*	**0.006**	1.17	*(0.71-1.96)*	0.537

Suicide ideation^3^	2.10	*(0.84-5.26)*	**0.094**	2.32	*(1.33-4.04)*	**0.003**

Suicide attempt^3^	1.37	*(0.97-1.94)*	**0.072**	0.50	*(0.08-3.38)*	0.480

**Nutritional health**						

Overweight	2.17	*(1.39-3.37)*	**< 0.001**	1.76	*(0.96-3.25)*	**0.069**

	***MD***_unadj_^***4***^			***MD***_mv_^***4***^		

Mean Height *(cm)*	-2.51	*(-3.71-1.30)*	**< 0.001**	-0.61	*(-1.75-0.54)*	0.298

**1**: in the past 30 days; **2**: in the past 7 days; **3**: in the past 12 months; **4: MD**: Mean Difference = (mean for LBC) - (mean for non-LBC);

**§**: Physically active represents children who were physically active for a total of at least 60 minutes per day, including all the time they spent in any kind of physical activity that increased their heart rate and made their breaths hard some of the time;

Adjusted models were fitted after controlling age, ethnicity, school type, boarder, parental education level, living with grandparents, house type, and computer-owning at home (for internet addiction only).

**Table 4:** Unadjusted and adjusted Odds Ratio (OR) of parental migration among girls (with either OR_unadj _or OR_mv _significant or marginally significant)

	females (non-LBC = ref)					
	
	OR_unadj_	*(95% CI) *	p-value	OR_mv_	*(95% CI)*	p-value
**Dietary intake**						

Ate vegetables ≥3 times/day^2^	*0.76*	*(0.57-1.00)*	**0.048**	*0.79*	*(0.57-1.08)*	0.143

Drank sweetened beverage ≥3 times/day^2^	*1.54*	*(1.05-2.25)*	**0.028**	*1.50*	*(0.95-2.37)*	**0.082**

**Physical activity & sedentary behaviours**						

Watched TV ≥4 hours/day^2^	*1.71*	*(1.24-2.36)*	**0.001**	*1.48*	*(1.01-2.17)*	**0.043**

**Internet use**						

Used internet^2^	*0.74*	*(0.55-0.99)*	**0.039**	*1.05*	*(0.72-1.54)*	0.791

**Tobacco & alcohol use**						

Ever smoked	*1.46*	*(0.98-2.16)*	**0.060**	*1.88*	*(1.19-2.96)*	**0.007**

Currently smoked^1^	*6.07*	*(2.02-18.24)*	**0.002**	*6.58*	*(2.00-21.69)*	**0.002**

Ever drank	*0.96*	*(0.72-1.28)*	0.786	*1.35*	*(0.97-1.87)*	**0.078**

Binge drinking^¶1^	*2.74*	*(1.49-5.02)*	**0.001**	*2.64*	*(1.30-5.37)*	**0.007**

**Emotional and mental issues**						

Felt unhappy most of the time or always^3^	*1.52*	*(1.08-2.15)*	**0.017**	*1.80*	*(1.20-2.68)*	**0.004**

Suicide plan^3^	*1.84*	*(0.81-4.18)*	0.140	*2.24*	*(0.91-5.56)*	**0.081**

Ideation of leaving home^3^	*1.34*	*(0.93-1.95)*	0.120	*1.50*	*(0.99-2.26)*	**0.055**

Attempt of leaving home^3^	*3.96*	*(1.46-10.73)*	**0.010**	*2.92*	*(0.79-10.81)*	0.109

**Nutritional health**	***MD***_unadj_^***4***^			***MD***_mv_^***4***^		

Mean Height *(cm)*	*-1.10*	*(-1.84--0.36)*	**0.004**	*-0.29*	*(-1.10-0.51)*	0.476

**1**: in the past 30 days; **2**: in the past 7 days; **3**: in the past 12 months; **4: MD**: Mean Difference = (mean for LBC) - (mean for non-LBC);

^¶^Binge drinking: ever drank at least 5 glasses of alcohol within 1 or 2 hours in the past 30 day;

Adjusted models were fitted after controlling age, ethnicity, school type, boarder, parental education level, living with grandparents, house type, and computer-owning at home (for internet use only).

#### Dietary behaviours and physical activity

Overall, most students had breakfast everyday (74.3%) and ate recommended portion of vegetables (59.1%). Less than one quarter of them consumed high-fat food (13.8%), sweetened snacks (23.4%) or sweetened beverages (18.1%) at least 3 times/day. Very few students felt hungry most of the time (1.3%) or drank milk at least 6 days/week (7.4%). Around half of children reported adequate physical activity levels (49.2%) although some of them were rather sedentary (19.0% watched TV, 15.5% did homework and 9.3% played e-games for at least 4 hours/day). After adjustment for the confounding factors, left behind boys were marginally less likely to eat breakfast everyday (OR_mv _= 0.75), but significantly less likely to be physically active (OR_mv _= 0.67) (Table 3). The female LBC were more vulnerable to excess drinking of sweetened beverages (OR_mv _= 1.50, P = 0.082) and excess TV watching (OR_mv _= 1.48, P = 0.043) (Table 4).

#### Internet use, tobacco use and alcohol use

More than half the students used internet in the past 12 months but few of them admitted to over-use (6.8%) or would be defined as internet addicted (5.8%). Compared to girls, boys were more likely to both smoke tobacco and drink alcohol (Table 2). Most boys had smoked (50.2%) or drunk alcohol (69.7%) in their lifetime and current smoking and binge drinking rates were 17.1% and 12.1% respectively among boys. In comparison to non-LBC, left behind boys were more likely to be internet addicted and to have ever smoked (OR_mv _= 1.39-1.77) after controlling for the confounders (Table 3). Left behind girls were at significantly higher risk for ever and current smoking and binge drinking before and after adjustment with confounders, with the highest OR_mv _of 6.58 found for currently smoking (Table 4). In addition, the nonsignificant OR_unadj _of parental migration for ever alcohol use among girls increased and reached marginal significance in the multivariate model (OR_mv _= 1.35) (Table 4).

#### Emotional and mental issues

The most common emotional problem among the students was unhappiness (15.3% felt unhappy always or most of the time in the past 12 months), and 9.4% expressed sadness. In addition, 8.7% and 14.1% had ever had ideations of suicide or leaving home respectively. More girls suffered from emotional issues than boys (Table 2). Left behind boys were more vulnerable to sadness and variables related to suicide and leaving home, but only ideas of suicide (OR_mv _= 2.23) achieved significance in the adjusted model (Table 3). Among girls, the unadjusted analyses revealed that LBC were more likely to report unhappiness and attempts to leaving home (OR_unadj _= 1.52-3.96), whilst the adjusted ORs showed that female LBC were at least marginally more likely to report unhappiness, ideas of suicide and thoughts of leaving home (OR_mv _= 1.50-2.24) (Table 4).

#### Nutritional health status

The mean values of height and weight were 160.5 cm and 47.9 kg for boys and 153.7 cm and 44.2 kg for girls (a mean age of 14.2 years for both genders) (Table 2). After controlling for potential confounders, the significant differences in mean height between LBC and non-LBC for both genders decreased and became nonsignificant (Tables 3 &4). Compared to girls, more boys had unhealthy weight (6.2% overweight, 1.7% obesity and 6.2% stunting) (Table 2). The significant higher risk for overweight among left behind boys slightly decreased and became marginally significant after adjustment for the confounders (OR_mv _= 1.76) (Table 3). Left behind girls were more vulnerable to obesity, but the ORs failed to achieve significance in both unadjusted and adjusted models (Table 4).

## Discussion and Conclusion

This cross-sectional study may be the first to explore the health impact of parental migration on the children they leave behind in rural China. Around one out of five children in the schools studied were identified as LBC and for nearly half of them both parents had migrated to work away from home. Our findings suggest that adolescent LBC are more likely to have a less healthy diet and lower rates of physical activity, to be more likely to be engaged in excessive computer-related activities, use substances such as smoking tobacco and to be overweight. In addition, the health impact of parental migration varies by gender. Girls are more likely to use substances and have emotional problems and express sadness, whilst left behind boys are more vulnerable to being overweight and addicted to the internet. One potential explanation could be the absence of parents but this needs further exploration since the impact of parental migration may be mitigated by having support from extended family members [14-16]. Another contributing factor could be the additional income from remittances from absent parents which contributes to greater affluence and higher standards of living [14-16].

The dynamics between parental migration and the impact on their children's health may contribute via indirect pathways to LBC health, for example through increased family income [14]. However, many students were unable to complete the part of the questionnaire asking about family income so we were not able to examine the relationship between family income and LBC behaviours. Housing type was used as a proxy for socioeconomic status and more LBC lived in multi-storey housing, possibly indicating greater family affluence in this part of China. National data has shown that income levels are higher for migrants than their rural counterparts [2]. In China, rising affluence has also been shown to increase unhealthy behaviours and contribute to overweight and obesity, since children with higher family incomes are more likely to have opportunities for unhealthy choices [34-36].

Our findings may corroborate previous reports that LBC are more likely to drop out of school [18,20]. The proportions of LBC decreased significantly from 23.3% in Year 1 junior secondary school students to 8.8% in Year 1 senior secondary school students. The decrease was much greater among left behind girls, suggesting that left behind girls tended to drop out earlier than their male counterparts [1,17]. These findings need further research but suggest that despite the better education of the majority of their parents and the assumed greater affluence, LBC adolescents might be at higher risk of not attaining their educational potential and also of health damaging behaviours.

Similar to previous studies, we observed a higher prevalence of smoking and drinking amongst rural adolescents compared to their urban counterparts [26,37]. Insufficient public awareness, lack of health education and promotion programmes, insufficient implementation of related regulations (e.g. prohibition of selling cigarettes to children under 18 and smoking in public places), and lack of support to help children to quit smoking and drinking in rural areas may contribute to the situation [38]. Evidence has shown that the majority of smokers start smoking before the age of 18, and the younger children are when they first try smoking, the more likely they are to become regular tobacco users and the less likely they are to quit [39]. In addition, we found that rural LBC are more vulnerable to substance use than non-LBC. Female LBC are 6 times more likely to currently smoke after adjustment for other risk factors. A systematic review on adolescent smoking in China revealed that parents' attitude towards the smoking behaviour of their children is the fourth leading determinant, following gender, teacher smoking, and peer smoking [40]. It may be that the lack of parents' presence is an explanation of the increased vulnerability to tobacco use among LBC. As a result, there is an urgent need to implement effective interventions in rural China with a focus on LBC to both stop adolescents from starting to smoke and to support them in quitting smoking. In addition, given more than 60% of Chinese adolescents live in rural areas, this would also contribute to decreasing the national smoking prevalence in the future.

Our study confirmed the evidence that emotional problems were more serious among adolescent LBC [18,20,21]. Stable family circumstance helps to promote psychological development of children and adolescents [4,20]. Parental migration results in LBC being cared for by other family members such as grandparents [20]. The differences in family role, education level, lifestyle between parents and other caretakers may contribute to an unfavorable environment for psychological development for adolescent LBC [21]. For instance, in China many migrating parents leave their children with grandparents; research has indicated that they may either spoil the children or fail to give them enough emotional care [18-21,23]. In our study, we found that living with grandparents is a risk factor for some health outcomes and such relationships achieved statistical significance or marginal significance in multivariate models for unhappiness (OR_mv _= 1.36, P = 0.091), suicide plan (OR_mv _= 3.12, P = 0.054), attempts to leave home (OR_mv _= 2.03, P = 0.088), as well as having felt hungry and skipping breakfast amongst boys (none for girls, data not shown). This may be due to grandparents being too old to have energy to give enough care to LBC or due to many grandparents receiving less formal education and taking a traditional "out-of-date" view on life, making it more difficult for LBC adolescents to adapt to a rapidly changing society and thereby contributing to the development of emotional problems [18-21,23].

Our study has several limitations. Firstly, it is a cross-sectional study, and therefore cannot be used to interpret cause-effect relationship. All of the demographic and behavioural data were self-reported by the students which may lend itself to reporting bias (such as under-reporting of stigmatising behaviours or over-reporting healthy behaviours). In order to minimize reporting bias, the students were asked to complete the questionnaire in class in their teachers' absence and anonymity of the study was stressed by investigators. The adopted YRBS and GSHS questionnaires have been shown to have acceptable validity and reliability [26,41,42]. Furthermore, it is unlikely that the reporting of health behaviours is systematically different between LBC and non-LBC, and therefore any bias may be not a major threat to our findings on the health impact of parental migration. Parental behaviours may play a role in the behaviour establishment among children. We did not collect information on this aspect in order to keep the questionnaire to an acceptable length. Given at least one of both parents of LBC are not living with them, the health impact of parental behaviours on adolescent LBC may be less than non-LBC. The type of parental migration (e.g. single or both parents' absence) may play a role in the health impact on LBC. However, our study has insufficient power to examine this hypothesis. Some factors such as the time length of parents being away from home and their communication with LBC may also contribute to the health impact of migration on LBC. Such information was not collected due to the limit of the length of questionnaire. Finally, this study was conducted only in a rural area of Southern China which may limit the generalisability of the results across China. In addition, this study was not able to cover the estimated 10% of rural children who are not enrolled in schools.

In summary, our study provides preliminary evidence that left-behind school children in rural Southern China are a vulnerable group who engage in higher rates of unhealthy behaviours and are likely to be overrepresented among both overweight and stunted groups. Parental migration appears to have a greater influence on girls in terms of substance use and mental health problems. Further studies are needed to explore the pathways and mechanisms by which the effect of parental migration impacts on LBC. Our findings have implications for prevention policies and programmes to promote healthy behaviours and improve health status among adolescent LBC in China and other developing countries facing similar internal migration issues.

## Competing interests

The authors declare that they have no competing interests.

## Authors' contributions

SG conceived and supervised the study and helped write the manuscript. YG completed the analyses and led the writing. LPL, JHK and JL led the revising. NC assisted with implementing the study. All authors have read and approved the manuscript.

## Pre-publication history

The pre-publication history for this paper can be accessed here:

http://www.biomedcentral.com/1471-2458/10/56/prepub
